# Pin1 Plays Essential Roles in NASH Development by Modulating Multiple Target Proteins

**DOI:** 10.3390/cells8121545

**Published:** 2019-11-29

**Authors:** Masa-Ki Inoue, Yusuke Nakatsu, Takeshi Yamamotoya, Shun Hasei, Mayu Kanamoto, Miki Naitou, Yasuka Matsunaga, Hideyuki Sakoda, Midori Fujishiro, Hiraku Ono, Akifumi Kushiyama, Tomoichiro Asano

**Affiliations:** 1 Department of Medical Science, Graduate School of Medicine, Hiroshima University, 1-2-3 Kasumi, Minamitnf-ku, Hiroshima City, Hiroshima 734-8551, Japan; b131831@hiroshima-u.ac.jp (M.-K.I.); nakatsu@hiroshima-u.ac.jp (Y.N.); ymmty@hiroshima-u.ac.jp (T.Y.); b151007@hiroshima-u.ac.jp (S.H.); mayu.hiro.094522@gmail.com (M.K.); mikinyuki@gmail.com (M.N.); 2 Center for Translational Research in Infection & Inflammation, School of Medicine, Tulane University, 6823 St. Charles Avenue, New Orleans, LA 70118, USA; ymatsunaga@tulane.edu; 3 Division of Neurology, Respirology, Endocrinology and Metabolism, Department of Internal Medicine, Faculty of Medicine, University of Miyazaki, 5200 Kihara, Kiyotake, Miyazaki 889-1692, Japan; hideyuki_sakoda@med.miyazaki-u.ac.jp; 4 Division of Diabetes and Metabolic Diseases, Nihon University School of Medicine, Itabashi, Tokyo 173-8610, Japan; fujishiro.midori@nihon-u.ac.jp; 5 Department of Clinical Cell Biology, Graduate School of Medicine, Chiba University, 1-8-1 Inohana, Chuo-ku, Chiba City, Chiba 260-8670, Japan; hono@chiba-u.jp; 6 Department of Pharmacotherapy, Meiji Pharmaceutical University 2-522-1 Noshio Kiyose, Tokyo 204-8588, Japan; kushiyamake@gmail.com

**Keywords:** Pin1, NASH, NAFLD, fibrosis, lipid, inflammation

## Abstract

Pin1 is one of the three known prolyl-isomerase types and its hepatic expression level is markedly enhanced in the obese state. Pin1 plays critical roles in favoring the exacerbation of both lipid accumulation and fibrotic change accompanying inflammation. Indeed, Pin1-deficient mice are highly resistant to non-alcoholic steatohepatitis (NASH) development by either a high-fat diet or methionine–choline-deficient diet feeding. The processes of NASH development can basically be separated into lipid accumulation and subsequent fibrotic change with inflammation. In this review, we outline the molecular mechanisms by which increased Pin1 promotes both of these phases of NASH. The target proteins of Pin1 involved in lipid accumulation include insulin receptor substrate 1 (IRS-1), AMP-activated protein kinase (AMPK) and acetyl CoA carboxylase 1 (ACC1), while the p60 of the NF-kB complex and transforming growth factor β (TGF-β) pathway appear to be involved in the fibrotic process accelerated by Pin1. Interestingly, Pin1 deficiency does not cause abnormalities in liver size, appearance or function. Therefore, we consider the inhibition of increased Pin1 to be a promising approach to treating NASH and preventing hepatic fibrosis.

## 1. Introduction

Non-alcoholic fatty liver disease (NAFLD) is characterized as hepatic steatosis that is not caused by factors such as significant alcohol consumption, chronic viral hepatitis and the side effects of medicines. In recent years, the number of NAFLD patients has been rising, showing trends similar to those of other diseases included in the metabolic syndrome category. In addition, approximately 10% of these NAFLD patients are categorized as suffering from nonalcoholic steatohepatitis (NASH), which is characterized by inflammatory steatosis and fibrosis, eventually progressing to liver cirrhosis and/or cancer [1,2]. NASH/NAFLD patients are currently estimated to comprise 20–40% of the global population [3,4,5,6]. Several clinical studies have suggested that dietary and exercise therapies for NASH/NAFLD improve biochemical indicators but do not ameliorate liver fibrosis [7,8].

Accordingly, various drugs, such as antidiabetic agents, have been studied as potential treatments for NASH/NAFLD. However, there are still no drugs that have gained general acceptance for treating NASH/NAFLD. Therefore, the elucidation of the molecular mechanisms underlying NASH development is essential for developing novel treatments [9,10,11,12,13,14], which should also be based on confirmed clinical evidence. Regarding these molecular mechanisms, the abnormalities impacting transcriptional factors, signal transduction molecules and post-translational modifications of these factors have been extensively investigated [3].

On the other hand, peptidyl-prolyl cis/trans isomerase Pin1 has the unique feature of associating with the domain containing the phospho-serine/proline or the phospho-threonine/proline motif, unlike the other prolyl isomerases (PPIases), e.g., FK506 Binding Protein and cyclophilin. Pin1 reportedly affects the function, stability and/or subcellular localization of its target proteins and thereby controls cellular proliferation, metabolism, fibrosis and inflammation [15]. The proline-directed serine/threonine phosphorylations are reportedly carried out by kinases such as cyclin-dependent kinases and mitogen-activated protein kinases (MAPKs) [16,17,18]. Therefore, the Pin1 expression level, as well as the phosphorylating activities mediated by serine/threonine-directed kinases, are important for the cis/trans isomerization at the proline residue of target proteins [19,20,21].

We consider Pin1 to be a key player in the development of NASH/NAFLD and discuss its roles in this review.

## 2. Prolyl Isomerase Pin1

Pin1 contains two functional domains which are connected by a flexible linker. Pin1 holds the WW domain (amino acids 1–39) in the N-terminal and the PPIase domain (amino acids 45–163) in the C-terminal [15,22,23,24]. In general, the WW domain associates with proline-rich sequences of target proteins. In the case of Pin1, however, phosphorylated Ser or Thr are required as the prior amino acid just before proline in order for binding to occur. The PPIase domain has the responsibility for isomerase activity.

Pin1 is expressed in various cells. In the liver, it has been experimentally shown that Pin1 is expressed at least in hepatocytes, Kupffer cells, and hepatic stellate cells (HSCs) [25,26]. As to liver diseases, Pin1 was first identified in liver cancer. Many studies have revealed a relationship between Pin1 and cancer. In fact, Pin1 strongly promotes both cancer development and growth through various pathways. In the cell cycle pathway, Pin1 increases cyclin D1 expression and thereby promotes cell cycle progression [27]. Pin1 also enhances the Notch signaling pathway. Pin1 activates Notch1 cleavage by the γ-secretase enzyme, thus increasing the release of the active intracellular domain and ultimately leading to the upregulation of both Notch1 transcriptional and tumorigenic activity [28]. In addition, the post-translational modification of Pin1 is thought to contribute to tumor formation in the liver. The phosphorylation of Pin1 Ser65 by Plk-1 is thought to stabilize Pin1 and promote carcinogenesis.

## 3. NASH/NAFLD

### 3.1. Genetic Factors Contributing to NASH/NAFLD

Many studies have clarified that the crosstalk between environmental and genetic background factors plays fundamental roles in NAFLD initiation and progression. Recent genome-wide association studies have unmasked the importance of genetic background. For example, the patatin-like phospholipase domain-containing 3 (PNPLA3) gene variant I148M is well recognized as a genetic risk factor for NASH and progressive hepatic injury [29].

### 3.2. “Two-Hit Theory” Versus “Multi-Hit Theory”

While the mechanism of NASH development has not been fully elucidated, the “two-hit theory has gained widespread recognition. According to this hypothesis, the “first hit” is the development of hepatic steatosis which is mediated mainly by lifestyle factors such as excessive caloric intake and inadequate physical exercise. The “second hit” is speculated to be induced by a variety of factors which lead to inflammation and fibrosis in the fatty liver. Oxidative stress, gut-derived endotoxins, some adipocytokines and free fatty acids (FFA) have been suggested as possible candidates for these factors [30,31]. However, recent findings raise the possibility that the two-hit theory is not fully applicable to the pathogenesis of NASH. This is because certain symptoms of inflammation are detectable before steatosis develops in some cases [32]. In addition, steatosis itself seems to be a favorable adaptation to excessive caloric intake, as triglyceride formation protects hepatocytes from lipotoxicity by “aggressive” lipids such as FFA which trigger reactive oxygen species (ROS) production and promote inflammation [33,34,35]. 

Furthermore, many factors including insulin resistance, dysregulation of FFA and adipokine secretion from adipose tissue and dysregulation of gut-derived signals via the alteration of gut microbiota and permeability appear to promote both steatosis and inflammation in parallel rather than sequentially. Based on these concepts, the “multiple parallel hit” theory appears to be more applicable than the “second hit” theory as an explanation for the pathogenesis of NASH [22,35].

**Figure 1:**
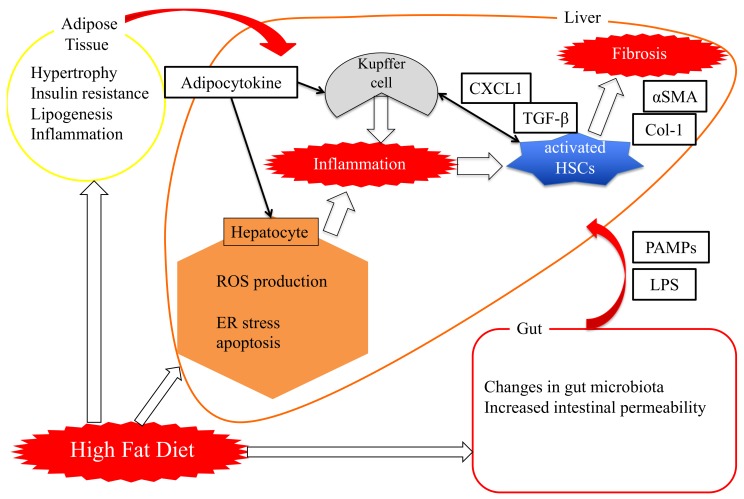
Physiological mechanisms of hepatic fibrosis development in NASH. Hepatic inflammation is enhanced by the influx of inflammatory substances from adipose tissue and the gut due to excessive calorie intake and a high-fat diet. Hepatic immune cells produce cytokines such as the tumor necrosis factor (TNF)α and activate quiescent HSCs, enhance the proliferation or survival of HSCs and cause the accumulation of lipid droplets and impairment of insulin sensitivity in hepatocytes. Activated HSCs induce hepatic fibrosis through the release of fibrotic factors. HSCs: hepatic stellate cells, PAMPs: Pathogen-associated molecular patterns. LPS: lipopolysaccharide. CXCL1: C-X-C Motif Chemokine Ligand 1. αSMA: α-smooth muscle actin. TGF-β: transforming growth factor β.

Pin1 expression in NASH model mouse livers was shown to be dramatically increased by either a methionine–choline-deficient diet (MCDD) or a high-fat diet (HFD). Importantly, Pin1 deficiency markedly suppressed the development of hepatic steatosis, inflammation and fibrosis observed in NASH mouse livers. Bone marrow transplantation experiments revealed the presence of Pin1 in both hematopoietic and non-hematopoietic cells to be essential for inflammation and lipid accumulation, respectively, with both contributing to NASH development [36]. Also, as described below in Figure 1, Pin1 has been shown to be involved in lipid metabolism, inflammation and fibrosis in the liver.

Taken together, these findings indicate upregulated hepatic Pin1 expressions to be essential for NASH development, making contributions to both inflammation and lipid accumulation, with Pin1 deletion or inhibition markedly mitigating NASH symptoms according to the “multi-hit” theory.

## 4. Role of Pin1 in the Pathogenesis of Hepatic Steatosis

Hepatic steatosis results from an imbalance between lipogenesis and fatty acid oxidation [30]. As described above, hepatic Pin1 expressions are increased by either an HFD or MCDD, and hepatic steatosis does not occur in Pin1 knockout (KO) mice [37]. These findings indicate Pin1 in the liver to be essential for the development of steatosis. 

First, insulin signaling plays critical roles in lipogenesis, by inducing the expressions of two rate-limited enzymes, acetyl CoA carboxylase 1 (ACC1) and fatty acid synthase (FASN) [38,39]. Pin1 reportedly enhances insulin signaling via its association with insulin receptor substrate 1 (IRS-1) and Akt [37,40]. Pin1 binds to IRS-1 and upregulates its tyrosine phosphorylation without altering IRS-1 protein levels. Indeed, Pin1 null mice were revealed to have impaired insulin sensitivity [37]. Moreover, Pin1 raises the level of Akt phosphorylation at Ser473 through the stabilization of the total Akt protein amount [39].

A recent report also clarified that Pin1 directly interacts with ACC1 and increases its protein stability [41]. Indeed, while Pin1 KO or knockdown markedly reduces the half-life of the ACC1 protein, Pin1 overexpression increases it. Similarly, the protein expression level of FASN is also increased by Pin1 without changing its mRNA level [42].

Third, Pin1 reportedly associates with the CBS domain in the γ subunit and reduces AMPKα subunit phosphorylation [43]. The reduction in AMPKα subunit phosphorylation mediated by Pin1 is likely due to the protective effect exerted by AMP or ADP, against dephosphorylation by protein phosphatase 2C (PP2C), being abolished. As a result, Pin1 negatively regulates AMPK activity [43]. AMPK, known as a major controller of lipid metabolism, directly phosphorylates ACC1 at the Ser79 site, and decreases both its activity and the productions of malonyl-CoA [44,45]. The reduction of malonyl-CoA indirectly upregulates fatty acid oxidation by abrogating the suppression of carnitine palmitoyltransferase 1 (CPT-1) [46,47]. In other words, AMPK inhibits lipogenesis and enhances fatty acid oxidation. Consequently, AMPK activation strongly suppresses lipid accumulation in the affected tissues.

**Figure 2:**
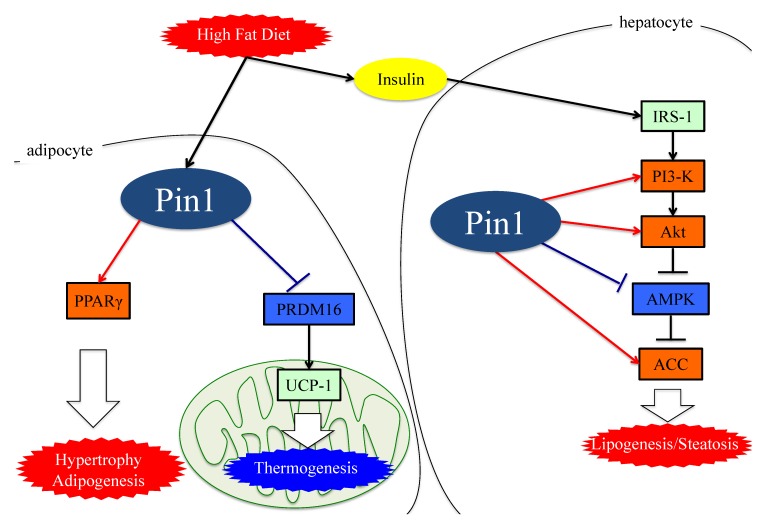
The functions of Pin1 in the molecular mechanism of lipogenesis in adipocytes. Either an HFD or MCDD increases Pin1 expressions in the liver and adipocytes. In adipocytes and hepatocytes, Pin1 enhances lipogenesis through an insulin-signaling-dependent mechanism by associating with IRS-1 and Akt, exerting AMPK-dependent and direct actions on lipid enzymes such as ACC1 and FASN. In the energy consumption pathway, Pin1 downregulates the expressions of thermogenic genes, such as UCP-1, and reduces O2 consumption. Pin1 also enhances adipocyte differentiation by activating transcription of PPARγ protein in adipocytes. IRS-1: insulin receptor substrate 1. ACC: acetyl CoA carboxylase. PRDM16: PR domain containing 16. UCP-1: Uncoupling protein 1. PPARγ: peroxisome proliferator-activated receptor γ.

Collectively, these observations indicate that Pin1 favors enhanced lipogenesis, functioning via at least three mechanisms; through an insulin-signaling-dependent association with IRS-1 and Akt, as well as exerting AMPK-dependent and direct actions on lipid enzymes such as ACC1 and FASN, as seen in Figure 2.

## 5. Essential Role of Adipose Pin1 in Obesity and NASH Development

Obesity is one of the risk factors for a fatty liver, though not all cases are obese. Recent reports have revealed that excessive lipid accumulation leads to adipocyte fibrosis [48]. Fibrotic adipocytes lose the ability to store triglycerides and ectopic fat accumulates in the affected livers. Accordingly, the amelioration of obesity rescues these livers from excessive fat storage. 

Insulin resistance with hyperinsulinemia are known to further promote obesity. Pin1 controls glucose metabolism through insulin signaling, AMPK and transcription of the enzymes regulating gluconeogenesis [38]. Indeed, Pin1 null mice exhibit impairments of both glucose and insulin tolerance [49]. In addition, Pin1 is a key player in the release of insulin, as Pin1 deletion in islets alleviates insulin release in response to high glucose [50]. In contrast, adipocyte-specific Pin1 knockout (KO) mice have lower body weights, with liver triglyceride and macrophage infiltration of epididymal white adipose tissue when fed a HFD [37].

Interestingly, adipocyte-specific Pin1 KO mice show an alleviation of both HFD-induced obesity and fatty liver development, indicating the amelioration of obesity to be linked to the prevention of NASH. Pin1 enhances adipocyte differentiation by activating the transcription of peroxisome proliferator-activated receptor γ (PPARγ) protein in adipocytes without affecting PPARγ protein levels [51]. The study also revealed PPARγ expression levels to be upregulated during adipogenesis and that Pin1 knockdown impairs adipogenesis. 

A recent investigation found that Pin1 is involved in the regulation of non-shivering thermogenesis in both brown and beige adipocytes. Pin1 interacts with the PR domain containing 16 (PRDM16), which is involved in the expression of the uncoupling protein (UCP-1), a major contributor to thermogenic programs. PRDM16, by associating with Pin1, is rapidly degraded through the ubiquitin–proteasome system, depending on the isomerase activity of Pin1. Indeed, Pin1 deletion in adipocytes elevates the expression of thermogenic genes and promotes O2 consumption [37]. 

Therefore, it has now become clear that not only hepatic but also adipose Pin1 is a major player in obesity development, contributing to the development of NAFLD or NASH, as shown in Figure 2.

## 6. Pin1 Promotes the Generation of ROS by Associating with NADPH Oxidase

Excessive free fatty acids (FFA) activate oxygen consumption in mitochondria. In the respiratory chain, which is activated in the presence of excessive FFA, complexes I and II are the main sources of mitochondrial ROS [52]. FFA present in excess also activates NADPH oxidase via protein kinase C (PKC) and produces ROS [53]. The NADPH oxidase (Nox) consists of six subcomponents (p47phox, p67phox, p40phox, Rac2, p22phox and gp91phox), in addition to Nox2 itself [54,55]. Although the mechanism of Nox2 activation is complex, it is essential that p47phox, phosphorylated by stimuli, be transferred to the membrane and then associate with p22phox.

Several studies have shown that Pin1 enhances superoxide production via NADPH oxidase activation [56,57]. The Toll-like receptor 7/8 (TLR7/8) agonists CL097 and TNF-α reportedly exert priming effects on N-Formylmethionyl-leucyl-phenylalanine (fMLF)-induced NADPH oxidase-mediated ROS production via p47phox translocation to the membranes. Pin1 binds to p47phox via phosphorylated Ser345 and then promotes p47phox translocation.

Increased ROS production in cells activates the NFE2-related factor 2 (Nrf2) and Forkhead box O (FoxO) pathways, functioning to both regulate and manage oxidative stress [58,59,60]. Nrf2 is a transcription factor also activated by phosphatidylinositol-5-phosphate (PtdIns5P), and is usually inactivated by binding to Kelch-like ECH-associated protein 1 (KEAP1) [58,61,62]. PtdIns5P levels are strictly regulated by phosphatidylinositol 5-phosphate 4-kinase (PIP4K) [62]. Stimulation with ROS oxidizes KEAP1 and this modification results in dissociation between Nrf2 and KEAP1. Consequently, Nrf2 which is translocated to the nucleus accelerates the transcriptions of antioxidant genes, such as heme-oxygenase1 [58,63].

In the Nrf2 pathway, Pin1 inhibits Nrf2 and PIP4K, which synthesize PtdInsP2 (4, 5) and remove PtdIns5P by phosphorylating it. A study on vascular smooth muscle cells showed that Pin1 overexpression increases the ubiquitination of Nrf2 and inhibits its nuclear translocation [64]. Another study showed that Pin1 and PIP4K co-localize in nuclear speckles and block the actions of PtdIns5P, which is the only known activator of Nrf2, by phosphorylating it [65].

## 7. Pin1 Enhances Inflammation

Inflammation and fibrotic change contribute to progression from NAFLD to NASH. Expressions of inflammatory cytokines are largely regulated by the pathways of the two transcription factors, nuclear factor-kB (NF-kB) and activator protein 1 (AP-1) [52,53,56]. Pin1 enhances both of these transcriptional pathways, as described below.

The family of inducible transcription factors NF-κB/Rel impacts cell survival, inflammation and the process of tumorigenesis. [66,67]. There are five structurally related NF-κB family proteins, including p50, p52, RelA (p65), RelB and c-Rel [68]. NF-κB exists in heterodimer or homodimer forms and is usually localized in the cytoplasm via interactions with IκB family members [69]. The NF-κB pathway activation begins with the inducible degradation of IκBα due to site-specific phosphorylation by the multi-subunit IκB kinase complex [70]. The detachment of IκBα induces the nuclear translocation of RelA/p50 heterodimers and initiates the induction of various pro-inflammatory genes [71]. Activated p65 and P50 bound to DNA are inactivated upon moving to the cytoplasm in response to rebinding with IκB [72,73]. Ryo et al. reported that Pin1-specific binding of p65 to the pThr254-Pro motif inhibits p65 from binding to IκBα, thereby enhancing p65 nuclear localization. Moreover, Pin1 exerts effects on p65 protein stability, since Pin1 deficiency reduces p65 protein levels. Accordingly, Pin1 regulates NF-κB pathway activation by controlling both the translocation and the protein stability of p65 [74,75]. Another study also found that Pin1 interacts with c-Rel and thereby promotes both nuclear translocation and transforming activity [76].

**Figure 3:**
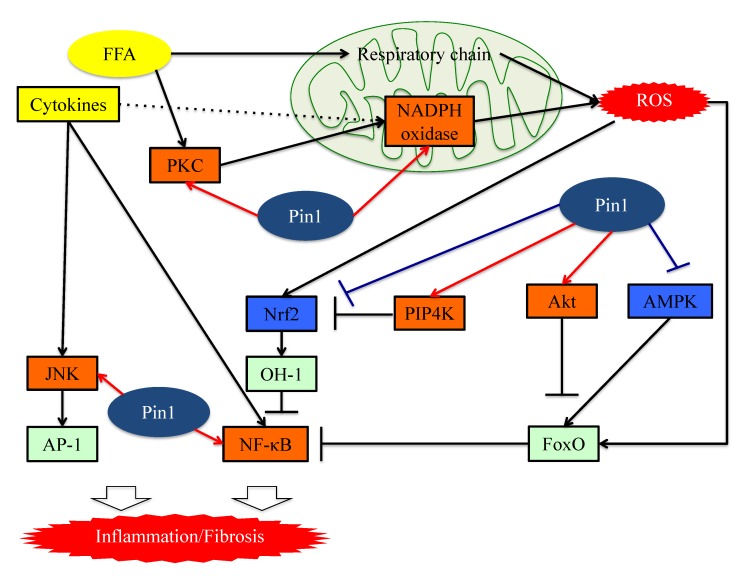
The functions of Pin1 in the molecular mechanism underlying inflammation in hepatocytes. Pin1 enhances superoxide production via NADPH oxidase activation and increases ROS production. In the downstream portion of this pathway, Pin1 inhibits the ROS resistant pathway and induces hepatic inflammation. Pin1 also activates the NF-κB and JNK-AP-1 pathways, thereby inducing hepatic inflammation. ROS: reactive oxygen species. FFA: Free fatty acid. FoxO: Forkhead box O. Nrf2: NFE2-related factor 2. NF-kB: nuclear factor-kB.

In the AP-1 signaling pathway, AP-1 transcriptional activity is regulated by c-Jun N-terminal kinase (JNK), a member of the mitogen-activated protein kinase (MAPK) group [77]. JNK activity is upregulated by phosphorylation of Thr and Tyr residues and these phosphorylations are a response to a variety of stressors such as oxidative stress or inflammatory cytokines [78,79]. Pin1 activates this pathway by interacting with phosphorylated JNK1 [78]. The bindings of Pin1 and JNK1 induce the interaction of JNK1 to c-Jun and activating transcription factor 2 (ATF2), which are downstream members of the AP-1 family. Pin1 also activates the AP-1 signaling pathway via upregulation of p70S6K activity [80] (Figure 3).

## 8. Pin1 Activates the Pathways Leading to Fibrosis

Yılmaz and colleagues raised the possibility that Pin1 concentrations in serum may well be exploited as markers for the detection of NASH and for determining advanced fibrotic scores for this disease [81]. The serum Pin1 levels were found to correlate with histopathological features in patients with NASH and to be independent predictors of advanced liver fibrosis. Furthermore, treatment with the Pin1 inhibitor juglone reportedly ameliorates drug-induced liver fibrosis [26]. The study that obtained this finding showed the Pin1 expressions in fibrotic livers to be upregulated, and that Pin1 regulates the expression of transforming growth factor-β (TGF-β)1, a fibrogenesis regulator, and the phosphorylation of Smad2/3, a key regulator of the fibrogenesis signaling pathway, in hepatic stellate cells (HSCs).

Numerous studies have confirmed the importance of the TGF-β-SMAD signaling pathway during the process of hepatic fibrosis. TGF-β is a ubiquitous factor which reportedly promotes fibrosis in various tissues [82,83]. TGF-β signaling occurs via both canonical Smad and non-Smad pathways [84]. TGF-β receptor I (TGF-βRI) phosphorylates the intracellular mediator receptor-regulated Smads (R-Smads), Smad2 and Smad3 [84,85,86]. Phosphorylated R-Smad is a dimer and the common mediator Smad (Co-Smad), also known as Smad4, is another member of the Smad family, and forms heterodimeric complexes in the cytosol [84,87]. The Smad complex accumulates in the nucleus and cooperates with other transcription factors to control gene transcription [84]. These Smad complex activities are negatively regulated by Smad6 and Smad7 which constitute a third class of Smad proteins, referred to as inhibitory Smad (I-Smad) [84].

Pin1 reportedly activates the TGF-Smad pathway and is thereby involved in fibrotic change [38,41,88,89]. The translational activity of TGF-β1 mRNA is regulated by various proteins. AU-rich element RNA-binding protein 1(AUF-1) binds to TGF-β1 mRNA and promotes its rapid exosome-mediated degradation [90]. Pin1 leads to the isomerization of hyper-phosphorylated AUF1 isoforms, and thereby reduces AUF1 binding to TGF-β1 mRNA and suppresses exosome-mediated mRNA decay [91]. Another study showed that Pin1 interacts with SUMOylated promyelocytic leukemia protein (PML), which acts as a nuclear translocation promoter and a TGFβ1 transcriptional regulator, increasing TGFβ1 mRNA expression and also, enhancing the TGFβ1-mediated Smad2/3 signaling pathway [92]. Pin1, as a part of the Smad pathway, interacts with both Smad2 and Smad3, thereby enhancing their phosphorylation and the resulting transcriptional activity [40,88,89].

In addition, Pin1-mediated isomerization of the inhibitory Smad6 MH2 domain may alter Smad6 functions such as nuclear localization signals and nuclear export signals. This isomerization increases phosphorylation, nuclear localization, and gene activation of Smad3 in response to TGFβ1 treatments and activates the TGF-β-Smad signaling pathway [93]. Several prior studies have confirmed that Pin1 knockdown or treatment with Pin1 inhibitors suppresses Smad phosphorylation and the expressions of fibrotic genes induced by the TGF-Smad pathway [38,89].

Signal transducers and activators of transcription (STATs) family pathways are also important for cytokine signaling and effects [94]. Leptin and IL-6 activate the STAT3 signaling pathway and increase HSC collagen mRNA expression [95]. Recent studies have shown that the complex of STAT3 and JunB can directly control activation of the COL1A2 enhancer [96,97]. In addition, STAT3 activates the TGF-Smad pathway. A study of dermal fibroblasts showed that activation of STAT3 by IL-6 results in TGFβ activation [98].

Stat3 transcriptional activity is regulated by Tyr-phosphorylation followed by dimerization and translocation from the cytosol to the nucleus. A breast cancer cell study demonstrated that Pin1 enhances STAT3 transcriptional activity by interacting with Ser727-phosphorylated STAT3 [99]. This study also revealed that Pin1 promotes STAT3-mediated epithelial–mesenchymal transition, thereby inducing liver fibrosis. 

**Figure 4:**
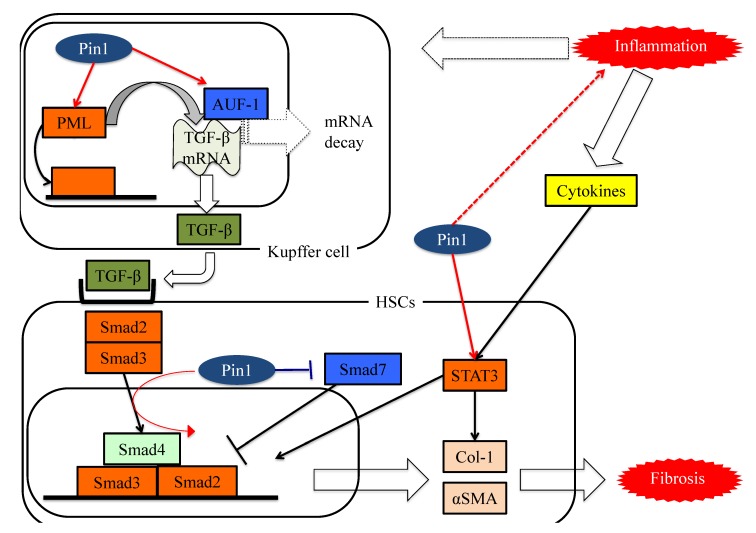
The functions of Pin1 in the molecular mechanism underlying fibrosis in HSCs. Pin1 activates TGF-β mRNA translation activity and the SMAD pathway in HSCs, which in turn activates extracellular matrix Col-1 and αSMA protein expression.

These studies showed Pin1 to be a key regulator of fibrosis and to have potential as a pharmacologic target for treating liver fibrosis, as seen in Figure 4.

## 9. Conclusions

Pin1 has been shown to regulate a variety of signal transduction pathways. In particular, Pin1 activates the pathways involved in not only lipid accumulation but also, inflammation and fibrosis. On the other hand, the results of analyses using Pin1 KO mice strongly suggest that Pin1 deficiency produces no abnormalities in liver size, formation or functions. Therefore, Pin1 inhibition is a potentially promising therapeutic method for NASH/NAFLD. In addition, it is clear that Pin1 inhibition has a marked effect on the suppression of carcinogenesis, and may exert a strong effect against hepatocellular carcinoma development. In addition, the serum Pin1 concentration is currently being studied for clinical application as a diagnostic marker for NASH/NAFLD. In conclusion, Pin1 plays a major role in all steps of the NASH process, including fat accumulation, fibrosis, and, perhaps ultimately, cancer development. However, the application of a Pin1 inhibitor, as NASH/NAFLD therapy, has not been adequately studied. Juglone (5-hydroxynaphthoquinone), epigallocatechin-3-gallate, cycloheptapeptidyl inhibitor, all-trans retinoic acid (ATRA) and arsenic trioxide (ATO) are known Pin1 inhibitors, but only juglone has been examined in NASH/NAFLD studies [41,100]. Since these inhibitors are not specific for Pin1, the development of a potent and highly selective Pin1 inhibitor, with minimal or no side-effects, for therapeutic use is eagerly awaited.
